# Analysis at the Atomic Level: The Atom Probe Field-Ion Microscope

**DOI:** 10.6028/jres.093.083

**Published:** 1988-06-01

**Authors:** M. K. Miller

**Affiliations:** Metals and Ceramics Division, Oak Ridge National Laboratory, Oak Ridge, TN 37831

## Introduction

The atom probe field-ion microscope (APFIM) is a unique analytical instrument that can analyze metals and semiconducting materials on the atomic scale. In recent years, the atom probe has developed into one of the most powerful instruments available for routine microstructural and microchemical analysis of materials. The types of investigations that have been performed have encompassed many diverse metallurgical subjects including phase transformations, segregation, diffusion, catalysis, and radiation damage [1].

## The Atom Probe Field-Ion Microscope

**Figure 1:**
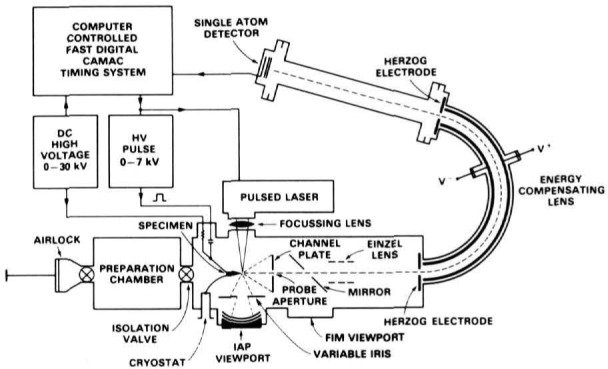
A schematic diagram of an atom probe field-ion microscope.

The atom probe combines an ultrahigh resolution field-ion microscope (FIM) with a mass spectrometer as shown in Figure 1. The FIM is capable of producing images of the surface of a specimen in which each distinct point on the image is an individual atom. The mass spectrometer is used to chemically analyze the specimen with single atom sensitivity for all elements.

After cooling to cryogenic temperatures, the field-ion image of the specimen surface is obtained by first introducing a small amount of image gas (e.g., neon) into the ultrahigh vacuum chamber and then applying a positive voltage to the specimen. At a certain voltage, dependent on the sharpness of the needle-shaped specimen and the image gas used, the field-ion image will appear on the channel plate and phosphor screen assembly. In order to make a chemical analysis of a specific region, such as a precipitate, the specimen is rotated until the image of that feature falls over the aperture in the channel plate and screen assembly. This aperture serves as the entrance to the mass spectrometer. The surface atoms of the specimen may then be removed from the specimen by increasing the voltage on the specimen by the process of field evaporation. This is generally done in practice by the superposition of a short high-voltage pulse onto the standing voltage already on the specimen. In the pulsed laser atom probe (PLAP) that is used for semiconducting materials, a short laser pulse is used instead. Whereas ionized atoms are removed from the entire specimen, only those whose trajectories pass through the aperture are analyzed in the mass spectrometer. The rate of field evaporation can be precisely controlled so that only a few atoms or many layers are removed from the specimen surface with each field evaporation pulse. The composition of the analyzed volume is determined by simply counting the number of atoms of each element in comparison to the total number. No calibrations or conversions are required, although some care must be taken in selecting experimental conditions to ensure that no preferential evaporation or retention occurs. The three-dimensional morphology of the phases or microstructures present in a specimen may also be reconstructed from a sequence of field-ion micrographs taken after successive field evaporation using video recording of the images. This persistence size technique also permits an accurate determination to be made of the size of small precipitates and their number density. Analysis of precipitates can be applied when the number density of the second phase is relatively large, i.e., > 10^20^ m^−3^. This type of analysis for less frequently occurring features is more difficult and time consuming because of the limited volume of material that is accessible for analysis in a field-ion specimen. The mass range of the atom probe is not restricted and the concentration of light elements may be readily determined. The negligible background noise level in the presence of the image gas enables chemical analysis to be made while viewing the field-ion image. The minimum detection level for trace elements is currently limited to approximately 10 appm because of the length of time required to collect sufficient ions.

In addition to the instrument outlined above, another variant known as the imaging atom probe (IAP) may be used to obtain elemental maps similar in form to those obtainable in an electron microprobe except with atomic spatial resolution [1].

## Examples

**Figure 2:**
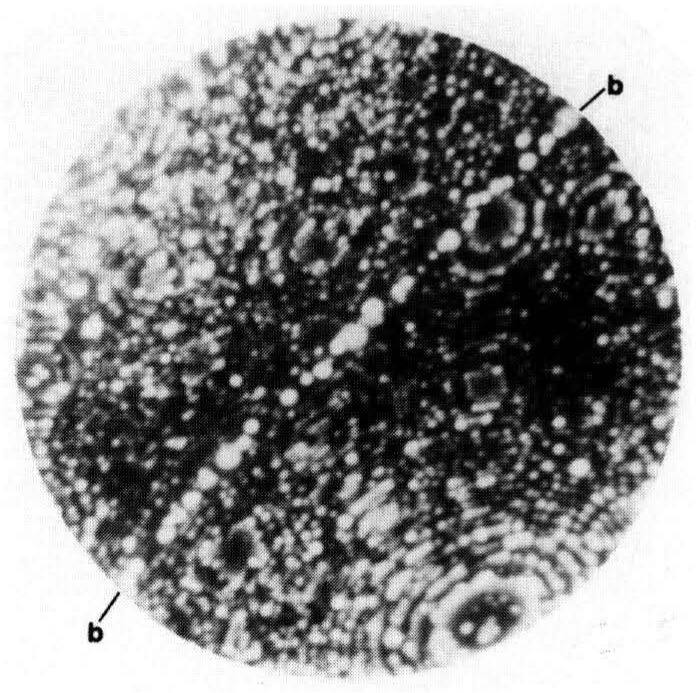
FIM micrograph of a boron-decorated boundary in rapidly solidified Ni-24.0 at. % Al-0.24 at. % B. Each bright spot is an individual boron atom.

The true uniqueness of the atom probe is clearly evident by its capability to chemically analyze a single atom. This type of analysis may be used to identify a segregant to a boundary as shown in figure 2. In this field-ion micrograph, a grain boundary in a boron-doped nickel aluminide specimen is decorated with bright spots. [2] The bright spots were identified as individual boron atoms by correlating the atom that produces the bright spot in the field-ion image with the atom that is collected in the mass spectrometer when it is field evaporated. This type of analysis is a specific example of selected area analysis and is used to determine the composition of small precipitates or compositional variations as a function of distance from features such as precipitates or boundaries. Precipitates smaller than 1 nm in diameter may be analyzed although the limited number of atoms that are available will control the statistical significance of the analysis.

**Figure 3:**
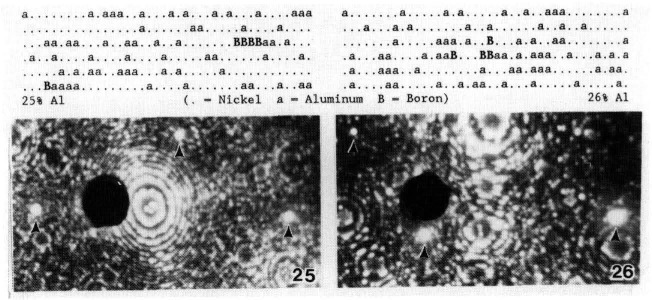
FIM micrographs and character plots of clusters in a Ni-25 at. % Al-0.48 at. % B and a Ni-26 at. % Al-0.48 at. % B. Each symbol in the character plots represents a single atom.

An alternative procedure for examining a specimen is to use random area analysis. This approach is used to analyze specimens where the selected area analysis is not possible or other information is desired. Situations where this method would be applied include clustering and cosegregation studies, high volume fraction microstructures such as found in systems that undergo spinodal decomposition, or systems where there is little or no contrast between the phases. In this method, a cylinder of atoms is collected from the specimen without regard to any feature in the image. In this way, the composition variations as a function of distance are measured. As material is removed, precipitates or clusters present in the specimen will eventually intersect the surface and those that emerge in the sampling volume will be analyzed. An example where this method was used [3] to detect small brightly-imaging boron clusters in other higher aluminum boron-doped nickel aluminide materials is shown in figure 3. The cluster size in this material was found to range from 2 to 7 boron atoms.
